# Randomized phase III study of gemcitabine, cisplatin plus S‐1 versus gemcitabine, cisplatin for advanced biliary tract cancer (KHBO1401‐ MITSUBA)

**DOI:** 10.1002/jhbp.1219

**Published:** 2022-08-09

**Authors:** Tatsuya Ioka, Masashi Kanai, Shogo Kobayashi, Daisuke Sakai, Hidetoshi Eguchi, Hideo Baba, Satoru Seo, Akinobu Taketomi, Tadatoshi Takayama, Hiroki Yamaue, Masahiro Takahashi, Masayuki Sho, Keiko Kamei, Jiro Fujimoto, Masanori Toyoda, Junzo Shimizu, Takuma Goto, Yoshitaro Shindo, Kenichi Yoshimura, Etsuro Hatano, Hiroaki Nagano, Kansai Hepatobiliary Oncology Group (KHBO)

**Affiliations:** ^1^ Department of Oncology Center Yamaguchi University Hospital Yamaguchi Japan; ^2^ Department of Cancer Survey and Gastrointestinal Oncology Osaka International Cancer Institute Osaka Japan; ^3^ Department of Medical Oncology Kyoto University Hospital Kyoto Japan; ^4^ Department of Gastroenterological Surgery Osaka University Graduate School of Medicine Osaka Japan; ^5^ Department of Frontier Science for Cancer and Chemotherapy Osaka University Osaka Japan; ^6^ Department of Gastroenterological Surgery Graduate School of Medicine, Osaka University Osaka Japan; ^7^ Department of Gastroenterological Surgery Graduate School of Medical Science, Kumamoto University Kumamoto Japan; ^8^ Department of Surgery Graduate School of Medicine, Kyoto University Kyoto Japan; ^9^ Department of Gastroenterological Surgery I Hokkaido University Graduate School of Medicine Hokkaido Japan; ^10^ Department of Digestive Surgery Nihon University School of Medicine Tokyo Japan; ^11^ Second Department of Surgery, School of Medicine Wakayama Medical University Wakayama Japan; ^12^ Department of Medical Oncology Tohoku University Hospital Sendai Japan; ^13^ Department of Surgery Nara Medical University Nara Japan; ^14^ Department of Surgery Kindai University Faculty of Medicine Osaka Japan; ^15^ Department of Gastroenterological Surgery Hyogo College of Medicine Hyogo Japan; ^16^ Department of Medical Oncology/Hematology Kobe University Hospital and Graduate School of Medicine Hyogo Japan; ^17^ Department of Surgery Toyonaka Municipal Hospital Osaka Japan; ^18^ Division of Gastroenterology and Hematology/Oncology Department of Medicine, Asahikawa Medical University Hokkaido Japan; ^19^ Department of Gastroenterological, Breast and Endocrine Surgery Yamaguchi University Graduate School of Medicine Yamaguchi Japan; ^20^ Medical Center for Clinical and Translational Research Hiroshima University Hospital Hiroshima Japan

**Keywords:** biliary tract cancer, cisplatin, gemcitabine, S‐1

## Abstract

**Background:**

Gemcitabine/cisplatin (GC) combination therapy has been the standard palliative chemotherapy for patients with advanced biliary tract cancer (BTC). No randomized clinical trials have been able to demonstrate the survival benefit over GC during the past decade. In our previous phase II trial, adding S‐1 to GC (GCS) showed promising efficacy and we aimed to determine whether GCS could improve overall survival compared with GC for patients with advanced BTC.

**Methods:**

We performed a mulitcenter, randomized phase III trial across 39 centers. Enrolled patients were randomly allocated (1:1) to either the GCS or GC arm. The GCS regimen comprised gemcitabine (1000 mg/m^2^) and cisplatin (25 mg/m^2^) infusion on day 1 and 80 mg/m^2^ of S‐1 on days 1–7 every 2 weeks. The primary endpoint was overall survival (OS) and the secondary endpoints were progression‐free survival (PFS), response rate (RR), and adverse events (AEs). This study is registered with Clinical trial identification: NCT02182778.

**Results:**

Between July 2014 and February 2016, 246 patients were enrolled. The median OS and 1‐year OS rate were 13.5 months and 59.4% in the GCS arm and 12.6 months and 53.7% in the GC arm, respectively (hazard ratio [HR] 0.79, 90% confidence interval [CI]: 0.628–0.996; *P* = .046 [stratified log‐rank test]). Median PFS was 7.4 months in the GCS arm and 5.5 months in the GC arm (HR 0.75, 95% CI: 0.577–0.970; *P* = .015). RR was 41.5% in the GCS arm and 15.0% in the GC arm. Grade 3 or worse AEs did not show significant differences between the two arms.

**Conclusions:**

GCS is the first regimen which demonstrated survival benefits as well as higher RR over GC in a randomized phase III trial and could be the new first‐line standard chemotherapy for advanced BTC. To exploit the advantage of its high RR, GCS is now tested in the neoadjuvant setting in a randomized phase III trial for potentially resectable BTC.

## Graphical abstract

Ioka and colleagues present the results of a phase III study comparing gemcitabine and cisplatin plus S‐1 (GCS) with gemcitabine and cisplatin for unresectable or recurrent biliary tract cancer. GCS demonstrated survival benefits and a higher response rate, and could be the new standard chemotherapy for advanced biliary tract cancer.

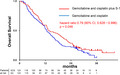

## 1 INTRODUCTION

Surgical resection is currently the only curative treatment for biliary tract cancer (BTC), including intra‐, and extra‐hepatic bile duct, gallbladder, and ampullary cancers. However, many cases go undiagnosed before reaching an advanced stage. Moreover, even when surgical resection can be performed, the recurrence rates of BTC are high, with 5‐year survival rates of <40%.^1^, ^2^, ^3^ Therefore, systemic therapy for patients with unresectable or recurrent disease largely relies on cytotoxic chemotherapy. Combination gemcitabine and cisplatin (GC) therapy has been the first‐line standard chemotherapy for advanced BTC since publication of the ABC‐02 study.^4^, ^5^ That study reported that GC versus gemcitabine alone significantly improved the median progression‐free survival (8.0 vs 5.0 months, *P* < .001) and median overall survival time (MST) (11.7 vs 8.1 months, *P* < .001).^5^ Similar results have been reported in the Japanese phase II study (BT‐22 study), which showed that GC therapy increased the MST from 7.7 to 11.2 months compared to gemcitabine monotherapy.^6^ The combination of GEM and S‐1 (GS), an oral fluoropyrimidine prodrug, has also been confirmed to be an effective therapy to treat advanced BTC in a phase III trial (FUGA‐BT).^7^, ^8^ Since GC was established as the standard of care, some phase III trials of alternatives have been conducted, all of which have had negative results in contrast to FUGA‐BT.^9^, ^10^, ^11^

We conducted a phase I/II clinical trial of the combination of GC and S‐1 therapy (GCS) with the aim of further improving the treatment outcomes for patients with unresectable BTC. After the recommended dose was determined in a phase I trial, a phase II trial evaluated the efficacy in 50 patients with advanced BTC and showed promising results with an MST of 16.2 months and a 1‐year survival rate of 60%.^12^, ^13^ On the other hand, the degree and incidence of adverse events (AEs) were similar to those of GC therapy. Therefore, we designed a randomized phase III study to evaluate the superiority of GCS over GC in terms of survival in patients with unresectable or recurrent BTC.

## 2 METHODS

### 2.1 Study design and patients

We performed a randomized, open‐label, phase III trial across 39 centers in Japan. Patients with advanced BTC who had experienced recurrence after surgery, or for whom curative surgery was not an option (unresectability was determined at the discretion of each institution), were eligible for the study if they met the inclusion criteria, which were the presence of adenocarcinoma or adenosquamous carcinoma of the biliary tract, intra‐ or extra‐hepatic cholangiocarcinoma, cancer of the gallbladder, or cancer of the ampulla of Vater (histologically or cytologically confirmed). Additionally, an Eastern Cooperative Oncology Group (ECOG) performance status of 0–2, age ≥20 years, adequate bone marrow (neutrophil count ≥1500/mm^3^, platelet count ≥100 000/mm^3^), liver (total bilirubin ≤3.0 mg/dL, aspartate aminotransferase [AST]/alanine aminotransferase [ALT] ≤150 IU/L), and renal functions (calculated creatinine clearance using Cockcroft and Gault formula ≥45 mL/min), and adequate oral intake.

Patients who had undergone prior chemotherapy or radiotherapy (except for adjuvant chemotherapy completed at least 6 months before enrollment) were not eligible for the study. Patients were also explicitly excluded if they had pulmonary fibrosis, interstitial pneumonia, severe heart disease, uncontrollable diabetes mellitus, active infection, severe drug hypersensitivity, mental disorder, watery diarrhea, moderate or marked pleural effusion or ascites necessitating drainage. Other serious medical conditions of individuals were deemed exclusionary at the discretion of the treating institute in each case. Women who were pregnant or lactating, or were within the childbearing age range for women (unless effective contraception was being used), were also excluded. Written informed consent was obtained from all the patients before commencement of the study and the institutional review boards or ethics committees at each site reviewed and approved the protocol (approval number of the representative institutional review board: C863‐2).

### 2.2 Randomization and masking

After the eligibility criteria were confirmed, registration with the KHBO Data Center was performed using a web‐based system or fax. The minimization method was employed to randomly assign patients in a 1:1 ratio to either the GC or GCS arm using the ECOG performance status (0 or 1 vs 2), the primary tumor (gallbladder vs nongallbladder), and history of primary tumor resection (recurrence vs unresectable) as randomization adjustment factors.

### 2.3 Procedures

The total duration of the study treatment period was 24 weeks. In the GC arm, 1000 mg/m^2^ of gemcitabine and 25 mg/m^2^ of cisplatin were infused on days 1 and 8 and repeated every 3 weeks. In the GCS arm, gemcitabine and cisplatin were administered intravenously at doses of 1000 and 25 mg/m^2^, respectively, on day 1, and oral S‐1 was administered orally twice a day for seven consecutive days repeated every 2 weeks. Doses of S‐1 were calculated according to body surface area (BSA) as follows: BSA <1.25 m^2^, 80 mg/day; 1.25 m^2^ ≤ BSA < 1.5 m^2^, 100 mg/day; and BSA ≥1.5 m^2^, 120 mg/day. Chemotherapy started and was repeated on day 1 if the neutrophil count was ≥1500/mm^3^, the platelet count was ≥100 000/mm^3^, total bilirubin was ≤3.0 mg/dL, AST/ALT was ≤150 IU/L and creatinine was ≤1.2 mg/dL. Moreover, only if there was no stomatitis/diarrhea of grade 2 or higher, and no fever (>38°C) due to infection or nonhematological toxicities of grade 3 or higher (except for abnormal blood test results not relevant to the study drugs). If the patient did not meet the above criteria, chemotherapy was postponed until recovery. S‐1 was discontinued if the patient met any of the following criteria during the treatment course: neutrophil count <1000/mm^3^, platelet count <75 000/mm^3^, total bilirubin >3.0 mg/dL, AST/ALT >150 IU/L, or if any of the aforementioned perquisite thresholds were breached. Additionally, if neutropenia (grade 4), thrombocytopenia (grade 4), febrile neutropenia, or nonhematological toxicity (grade 3) associated with gemcitabine occurred, the subsequent gemcitabine dose was reduced to 800 mg/m^2^. If further toxicity occurred with the reduced dose, it was further reduced to 600 mg/m^2^. If further dose reduction was necessary, the subsequent gemcitabine dose was reduced by 20%. If diarrhea, stomatitis, anorexia, nausea, or fatigue (grade 3) associated with S‐1 occurred, the dose of S‐1 was reduced in the subsequent cycle as follows: 80/60, 100/80, or 120/100 mg/day (before/after). If further reduction of S‐1 was necessary, the dose of S‐1 was reduced in the subsequent cycle as follows: 60/50, 80/60, or 100/80 mg/day (before/after). If further reduction of S‐1 was necessary, patients were withdrawn from the study. Cisplatin was suspended until recovery if the patient met any of the following criteria during the treatment course: neuropathy (grade 2 or higher) or hearing disturbance associated with cisplatin. No dose re‐escalation for each drug was permitted. The protocol treatment was halted before the full 24‐week term only if any of the following occurred: deterioration of general condition due to disease progression, unacceptable or repeated treatment‐related toxicity, a > 6‐week delay of the schedule due to treatment‐related toxicity, patient refusal, or tumor response allowing potential curative resection.

Pretreatment evaluation included obtaining the patient's medical history and performing a physical examination, imaging tests using contrast‐enhanced computed tomography (CT) or magnetic resonance imaging (MRI), blood tests, electrocardiography, and chest radiography. Physical examinations and blood tests were scheduled on day 1 of each course. Carcinoembryonic antigen and carbohydrate antigen 19‐9 were measured at the time of enrollment in the study and once monthly thereafter. Toxicity was evaluated using the Common Terminology Criteria for Adverse Events version 4.0. In patients with measurable target lesions, the objective response rate was assessed with no central review according to the Response Evaluation Criteria in Solid Tumors (RECIST) version 1.1,^14^ and CT or MRI were planned every 12 weeks after the initiation of treatment during protocol treatment. Additional imaging tests were performed if clinically indicated or at the discretion of the treating physician.

### 2.4 Outcomes

The primary endpoint was overall survival (OS). The secondary endpoints were progression‐free survival (PFS), overall response rate, and safety. OS was defined as the time from the date of registration to death from any cause. PFS was defined as the time from the date of registration to tumor progression or death from any cause whichever came first. Patients who did not die or did not have disease progression at the time of this analysis were censored at the date of their last follow‐up. The overall response rate was defined as the proportion of patients with the best, unconfirmed overall response of complete response or partial response in patients with measurable lesions, and the disease control rate was defined as the proportion of patients with complete response, partial response, or stable disease.

### 2.5 Statistical analysis

We sought to give the trial 80% power to detect a significant difference in the primary outcome between treatment groups with an overall one‐sided type I error rate of 0.05. We calculated that a sample size of 234 patients would be required, assuming a hazard ratio for death of 0.71, a 1‐year survival rate of 43% in the GC, and 55% in the GCS arm, an enrollment period of 3 years and a follow‐up period of 2 years. Considering the possibility of ineligible and untreated patients, the target number of enrolled patients was set at 240.

All efficacy endpoints were primarily assessed in the full analysis set (FAS) following the intention‐to‐treat principle, and safety was assessed in all treated patients. OS and PFS were analyzed using the Kaplan–Meier method, and comparisons between groups were conducted using the stratified log‐rank test. Stratified Cox proportional hazards models were used to estimate hazard ratios and corresponding 95% confidence intervals (CIs). Randomization adjustment factors were used in all the stratified analyses. The overall response and disease control rates were compared using Pearson's chi‐square test. All statistical analyses were performed using SAS (version 9.4; SAS Institute).

### 2.6 Role of the funding source

The funder of the study had no role in the study design, data collection, data analysis, data interpretation or writing of the report. The central data manager (HO), chief investigator (MK), associated investigators (TI, EH, and HN), and the trial statistician (KY) had full access to all the data. The decision to submit for publication was made after discussion within the trial management group. The corresponding author and chief investigator had full access to all the data in the study and had final responsibility for the decision to submit for publication.

## 3 RESULTS

**FIGURE 1:**
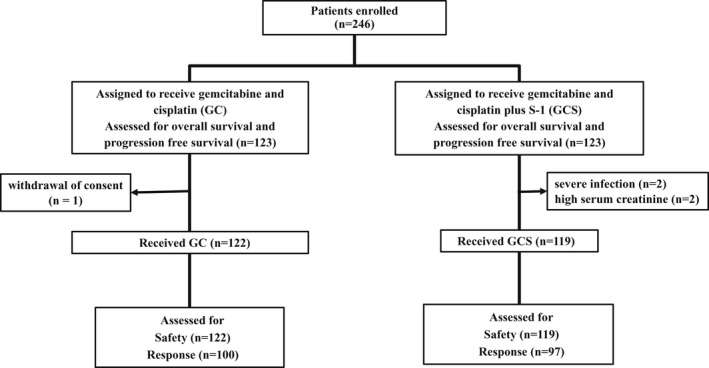
Schematic outline of the design parameters, metrics and observed quantities of the trial.

From July 9, 2014 to February 4, 2016, 246 patients were enrolled and randomized to receive either GC (n = 123) or GCS (n = 123) (Figure 1). One patient in the GC group withdrew consent and did not receive the assigned treatment. Four patients in the GCS group did not receive chemotherapy because of severe infection or high serum creatinine levels.

The baseline characteristics were well balanced between the groups (Table 1). The cutoff date for primary analysis was April 16, 2018. The median follow‐up time was 15.6 months.

**TABLE 1:** Baseline characteristics in randomly assigned patients

Factors	Gemcitabine and cisplatin	Gemcitabine and cisplatin plus S‐1
	(n = 123)	(n = 123)
Age (y)	68 (40–84)	68 (39–81)
Gender		
Male	66 (53.7)	68 (55.3)
Female	57 (46.3)	55 (44.7)
ECOG performance status		
0‐1	121 (98.4)	123 (100)
2	2 (1.6)	0 (0)
Measurable lesions		
No	22 (17.9)	25 (20.3)
Yes	101 (82.1)	98 (79.7)
Primary tumor site		
Gall bladder	40 (32.5)	42 (34.1)
Extra‐hepatic bile duct	38 (30.9)	43 (35.0)
Perihilar	19	26
Distal	19	17
Intrahepatic bile duct	43 (35.0)	35 (28.5)
Ampullary	2 (1.6)	3 (2.4)
Disease stage at entry		
Unresectable	93 (75.6)	91 (74.0)
Locally advanced	32 (26.0)	18 (14.6)
Metastatic	61 (49.6)	73 (59.3)
Recurrent	30 (24.4)	32 (26.0)

*Note*: Data are median (range), or number (%).

Abbreviation: ECOG, Eastern Cooperative Oncology Group.

Patients in the GC and GCS groups completed a median of six cycles (range 1–8 cycles) and nine cycles (range 1–12 cycles), respectively. The 24‐weeks protocol treatment was completed in 45 patients (36.6%) in the GC group and 60 patients (48.8%) in the GCS group. Main reasons for discontinuation of treatment were disease progression (48 [39%] of 123 patients in the GC group vs 38 [31%] of 123 patients in the GCS group), AEs (15 [12%] vs 11 [9%]), and patient's request (9 [7%] vs 4 [3%]). In the GC group, relative dose intensity (RDI) of gemcitabine and cisplatin were 75.1% and 78.3%. In the GCS group, RDI of gemcitabine and cisplatin, S‐1 were 83.1% and 86.0%, 81.1%.

**FIGURE 2:**
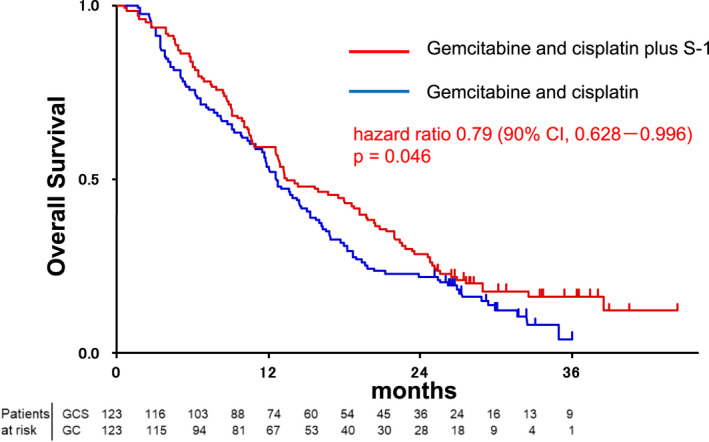
Overall survival of patients in the GC (blue) and GCS (red) arms of the study.

**FIGURE 3:**
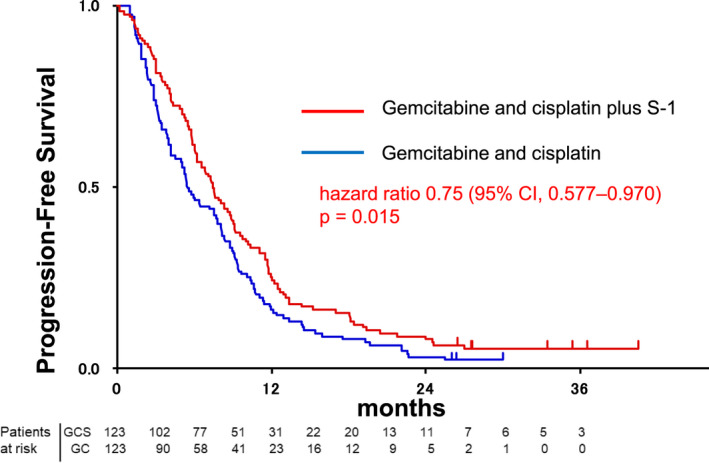
Progression‐free survival of patients in the GC (blue) and GCS (red) arms of the study.

**FIGURE 4:**
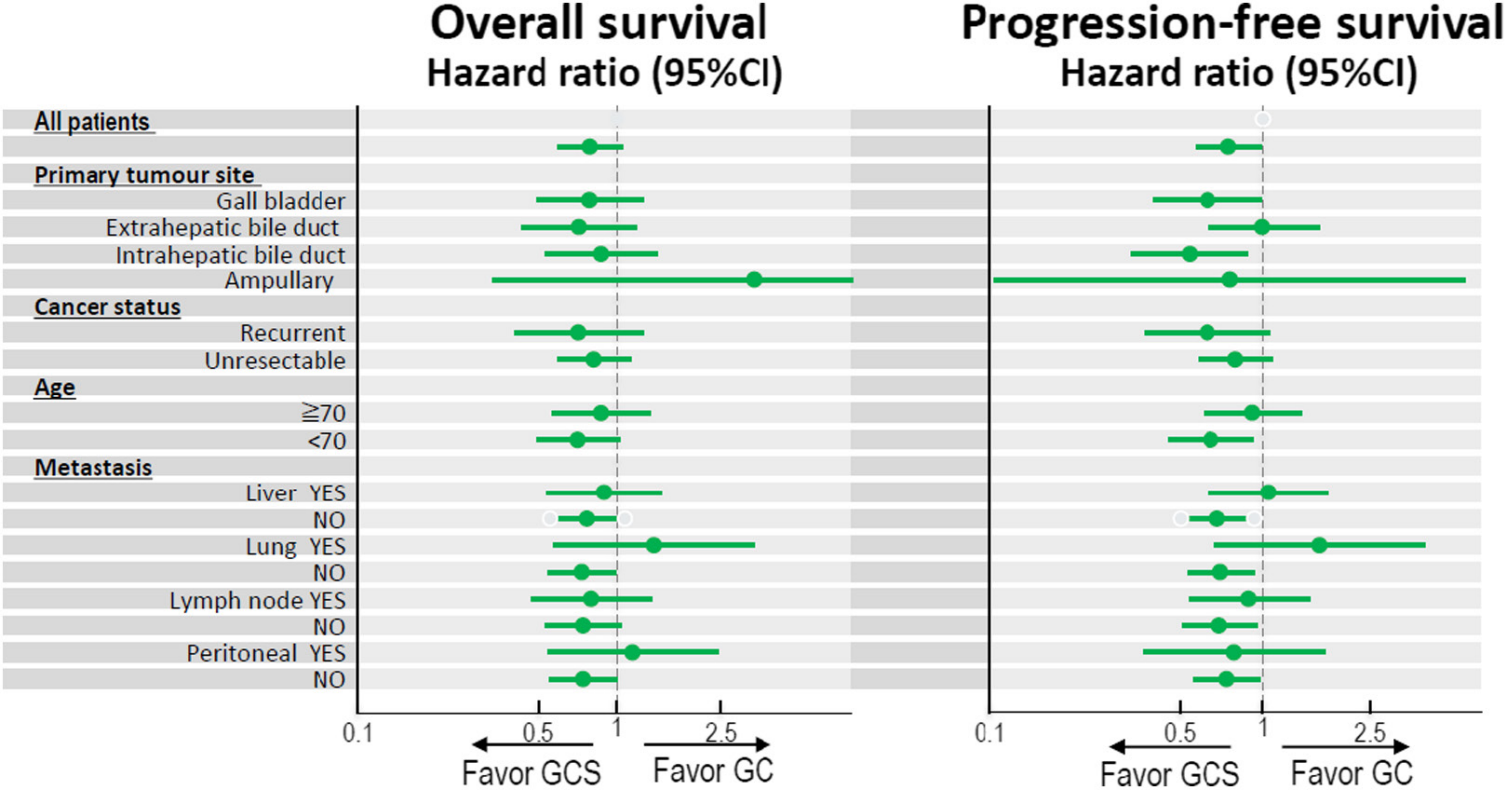
Subgroup analyses of overall survival and progression‐free survival.

At the time of analysis, 236 (96%) patients had experienced disease progression or died (120 [98%] of 123 patients in the GC group vs 116 [94%] of 123 patients in the GCS group). In the intention‐to‐treat population, median OS (Figure 2) and 1‐year OS was 12.6 months (95% CI: 10.9–14.6) and 53.7% (95% CI: 44.5–62.0) in the GC group and 13.5 months (12.6–18.8) and 59.4% (50.1–67.4) in the GCS group, respectively (HR 0.791, 90% CI: 0.628–0.996; *P* = .046). The superiority of GCS was statistically proved in this phase III trial. The median PFS (Figure 3) was 5.5 months (95% CI: 4.2–7.7) in the GC group and 7.4 months (6.2–8.9) in the GCS group (HR 0.748, 95% CI: 0.577–0.970; *P* = .015). Subgroup analysis of OS and PFS favored GCS compared with GC in most subgroups (Figure 4). In total, 194 patients were assessable for response (100 in the GC group and 94 in the GCS group). GCS was significantly better than GC in the analysis of objective response (41.5% vs 15.0%, *P* < .001; Table 2). The disease control rate of GCS was also significantly higher than that of GC (78.7% vs 62.0%, *P* = .0066). Three CRs and 36 PRs were recorded in the GCS group compared to one CR and 14 PRs in the GC group. The median duration of response was 5.49 months (95% CI: 3.05–8.48) in the GC group and 5.95 months (4.10–6.90) in the GCS group. The waterfall plot also demonstrates that GCS provides highly effective tumor shrinkage (Figure S1).

**TABLE 2:** Best tumor response by RECIST 1.1

	Gemcitabine and cisplatin (n = 100)	Gemcitabine and cisplatin plus S‐1 (n = 94)
Complete response	1 (1)	3 (3)
Partial response	14 (14)	36 (38)
Stable disease	47 (47)	36 (38)
Progressive disease	31 (31)	15 (16)
Not evaluable	7 (7)	4 (4)

*Note*: Data are number (%).

Abbreviation: RECIST, Response Evaluation Criteria in Solid Tumors.

Overall, 77 (63%) of 122 patients in the GC group and 71 (60%) of 119 patients in the GCS group received second‐line therapy after disease progression. The most frequently used agents were S‐1 monotherapy and gemcitabine monotherapy (in 45 [58%] and 22 [29%] of 77 patients in the GC group and 22 [31%] and 9 [13%] of 71 patients in the GCS group, respectively). Three patients in the GCS group underwent conversion surgery, but not in the GC group.

Both treatments were generally well tolerated. Neutropenia, thrombocytopenia, raised liver enzymes, fatigue and nausea were the most common toxic effects in both groups (Table 3). Diarrhea, stomatitis, and rash were more frequent in the GCS group than in the GC group. In contrast, sensory neuropathy was more frequent in the GC group than in the GCS group. However, these side effects were, in most cases, transient and easily manageable. The most common grade 3–4 AE was neutropenia in both groups. One patient in each group died of treatment‐related causes during the study period.

**TABLE 3:** Adverse events

	Gemcitabine and cisplatin (n = 122)		Gemcitabine and cisplatin plus S‐1 (n = 119)		*P*‐value
	Any grade	Grade 3–4	Any grade	Grade 3–4	
Neutropenia	97 (80)	58 (48)	92 (77)	47 (39)	NS
Anemia	27 (22)	18 (15)	24 (20)	10 (8)	NS
Thrombocytopenia	106 (87)	26 (21)	110 (92)	11 (9)	NS
Febrile neutropenia	5 (4)	5 (4)	6 (5)	6 (5)	NS
AST increased	95 (78)	25 (20)	89 (75)	18 (15)	NS
ALT increased	86 (70)	19 (16)	82 (69)	15 (13)	NS
Creatinine increased	45 (37)	0 (0)	32 (27)	1 (1)	NS
Fatigue	81 (66)	9 (7)	80 (67)	6 (5)	NS
Nausea	62 (51)	2 (2)	61 (51)	2 (2)	NS
Biliary tract infection	21 (17)	20 (16)	20 (17)	20 (17)	NS
Diarrhea	17 (14)	2 (2)	29 (24)	3 (3)	.037
Stomatitis	16 (13)	0 (0)	33 (28)	3 (3)	.0053
Sensory neuropathy	14 (11)	0 (0)	4 (3)	1 (1)	.019
Rash	9 (7)	3 (2)	27 (23)	1 (1)	.0012

*Note*: Data are number (%).

Abbreviations: ALT, alanine aminotransferase; AST, aspartate aminotransferase; NS, not significant.

## 4 DISCUSSION

In this phase III study, we found that OS in patients with advanced BTC was significantly improved with GCS compared with GC, meeting the primary endpoint. Our study demonstrated additional benefits in terms of PFS, RR with manageable safety profile. Therefore, GCS can be considered an alternative standard treatment option for advanced BTC.

There was clearly a plateau in the right tail of the survival curve in the GCS. GCS was associated with significant improvement of PFS and RR, which supported the results of improving OS.

The RR was almost tripled with GCS compared to GC. Although GC or GCS therapy was conducted for unresectable cancers due to locally advanced or metastatic disease, some patients could undergo resection as a conversion surgery.^15^, ^16^ Conversion surgery has been reported to have potential benefits for patient survival.^17^, ^18^, ^19^ In our previous phase I/II study, we experienced an effective case of conversion surgery. One patient with unresectable intrahepatic BTC received 12 cycles of GCS and had a PR. The patient then underwent conversion surgery. Surprisingly, histopathological examination revealed no residual tumor, resulting in a complete pathological response.^16^ Also, in this study, three cases of conversion surgery were performed in the GCS group, and it is possible that the high tumor shrinkage effect led to conversion surgery.

The proportions of diarrhea, stomatitis, and rash in all grades were more frequent in the GCS group than in the GC group. In contrast, sensory neuropathy of all grades was more frequent in the GC group than in the GCS group. The incidence of grade 3–4 toxicities in the GCS group was comparable to that in the GC group. The overall toxicity was generally manageable despite administration of the three cytotoxic drugs. We believe that the biweekly schedule largely contributed to the safety of this regimen.

The proportion of patients with poor prognostic factors such as gallbladder cancer or recurrent disease was 33 and 25%, respectively, which were comparable with those published studies including the ABC‐02 study (36 and 21%, respectively) and the FUGA‐BT study (39 and 21%, respectively).^5^, ^6^, ^8^, ^13^, ^20^ This suggests that our study cohort included patients with BTC who received chemotherapy in daily clinical practice rather than selected patients with favorable characteristics.

One caveat of our study was that it included only patients of Asian extraction. The pharmacokinetics and pharmacodynamics of S‐1 are known to differ between Caucasian and Asian patients.^21^, ^22^ With regard to these differences, we speculate that GCS therapy may not yield the same degree of improvement in patient outcome with respect to GC for Caucasians. A regimen using capecitabine instead of S‐1 would potentially prove effective in Caucasians.

In summary, GCS is the first regimen to have demonstrated survival benefits as well as higher RR over GC in a randomized phase III trial and could be the new first‐line standard chemotherapy for advanced BTC. To exploit the advantage of its high RR, GCS is now tested in the neoadjuvant setting in a randomized phase III trial for potentially resectable BTC.

## CONFLICT OF INTEREST

TI has received personal fees from Incyte, Chugai, Yakult Honsha, Taiho Pharmaceutical, Ono Pharm, Servier, Daiichi Sankyo, Nihon Zouki, Eli‐Lilly, Otsuka, Novartis, AstraZeneca and Abbott. MK has received personal fees from Chugai. MT has received personal fees from Ono. KY has received personal fees from Ohara, Nihon‐Shinyaku, Sysmex, Chugai Pharma, Eli‐Lilly, Astra Zeneca, Otsuka, Novartis, Eisai, Nihon‐Kayaku, Boehringer Ingelheim, Taiho and Pfizer. EH has received personal fees from Eli‐Lilly and Taiho. The other authors declare that they have no competing interest.

## Supporting information


Figure S1
