# Supplementary figures and images for: Randomized phase III study of gemcitabine, cisplatin plus S‐1 versus gemcitabine, cisplatin for advanced biliary tract cancer (KHBO1401‐ MITSUBA)
Source: J Hepatobiliary Pancreat Sci. 2022 Aug 9;30(1):102–10. doi: 10.1002/jhbp.1219 (PMC10086809; doi:10.1002/jhbp.1219)

## Slide 1
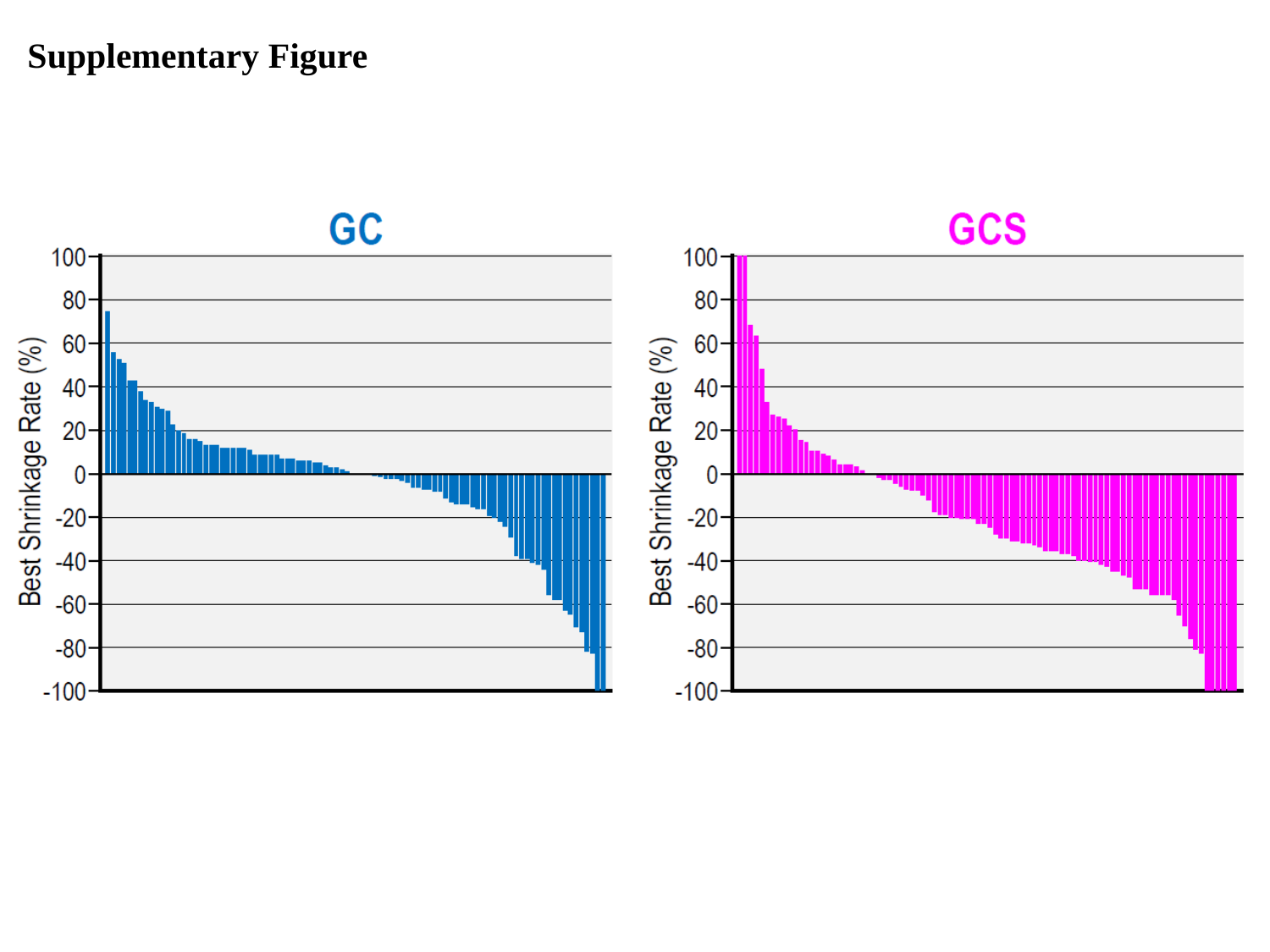

Supplementary Figure

Supplement: Supplementary file 1 — Figure S1 [file JHBP-30-102-s001.pptx]
